# Differentiating small intestinal stromal tumors from primary small intestinal lymphomas using contrast-enhanced CT and texture analysis: a diagnostic study

**DOI:** 10.3389/fonc.2025.1701049

**Published:** 2025-10-24

**Authors:** Zhihua Li, Yiying Wang, Da Xi, Jian Wang, Haiyang Lan, Xiaoning He, Na Zhao, Juan Xiao, Naiwen Mu, Jianlong Li, Lincheng Liu, Guanghui Yu

**Affiliations:** ^1^ Department of Radiology, People’s Hospital of Juxian, Rizhao, China; ^2^ Department of Special Inspection, Qingdao Women and Children’s Hospital, Qingdao, China; ^3^ Department of Radiology, People’s Hospital of Rizhao, Rizhao, China; ^4^ School of Radiology, Shandong First Medical University and Shandong Academy of Medical Sciences, Tai’an, China; ^5^ Department of Radiology, The Second Affiliated Hospital of Shandong First Medical University, Tai’an, China

**Keywords:** intestinal tumor, stromal tumor, lymphoma, CT, texture analysis

## Abstract

**Purpose:**

To assess the diagnostic performance of morphological features combined with contrast-enhanced computed tomography (CECT) texture analysis in differentiating small intestinal stromal tumors (SISTs) from primary small intestinal lymphomas (PSILs).

**Methods:**

This retrospective study included 77 patients with pathologically confirmed SISTs and 52 patients with PSILs who underwent CECT. Clinical data (age, sex, symptoms) and CT morphological features (tumor location, growth pattern, enhancement, etc.) were analyzed. Texture parameters (entropy, contrast, homogeneity, etc.) were extracted using 3D Slicer software (version 5.6.2; https://www.slicer.org/). Statistical comparisons were performed using Student’s t-test or Mann–Whitney U test. Receiver operating characteristic (ROC) curve analysis was used to evaluate diagnostic efficacy.

**Results:**

Compared with PSILs, SISTs exhibited significantly higher entropy (6.21 ± 0.45 vs. 5.12 ± 0.38, P < 0.001) and contrast (45.6 ± 12.3 vs. 28.7 ± 9.4, P = 0.003), but lower homogeneity (0.32 ± 0.08 vs. 0.51 ± 0.11, P = 0.002). The combined model integrating CECT morphological and texture features achieved an AUC of 0.927 (95% CI: 0.879–0.975), outperforming CECT features alone (AUC = 0.847).

**Conclusion:**

The integration of CECT morphological features and texture analysis enhances the differentiation of SISTs from PSILs, offering a valuable tool for improving preoperative diagnostic accuracy and guiding clinical decision-making in intestinal tumors.

## Introduction

Small intestinal stromal tumors (SISTs) and primary small intestinal lymphomas (PSILs) are two distinct malignancies with markedly different biological behavior and management strategies. SISTs are mesenchymal neoplasms with malignant potential, accounting for approximately 30% of small intestinal stromal tumors (1). Although surgical resection is the standard treatment for localized disease (2), high-risk cases remain prone to recurrence and metastasis despite advances in targeted therapies such as imatinib (3–6).

In contrast, PSILs represent a heterogeneous group of lymphoid malignancies, comprising 30-40% of all extranodal lymphomas (7). Their clinical presentation, prognosis, and treatment - which is primarily chemotherapy or radiotherapy - differ substantially from those of SISTs (8–11). Accurate preoperative distinction between these entities is therefore critical for treatment selection and prognostic assessment.

Conventional computed tomography (CT) is widely used for evaluating small intestinal tumors, but the morphological features of SISTs and PSILs often overlap, limiting diagnostic accuracy (12, 13). Radiomics, particularly CT texture analysis (CTTA), enables high-throughput quantification of tumor heterogeneity, providing information beyond visual interpretation (14–17). While CTTA has shown promise in oncologic imaging (18), its application in differentiating SISTs from PSILs has not been systematically investigated.

This study aimed to assess the value of contrast-enhanced CT morphological features combined with texture analysis in differentiating SISTs from PSILs, with the goal of improving preoperative diagnostic accuracy and guiding clinical decision-making.

## Materials and methods

### Study population

**Figure 1:**
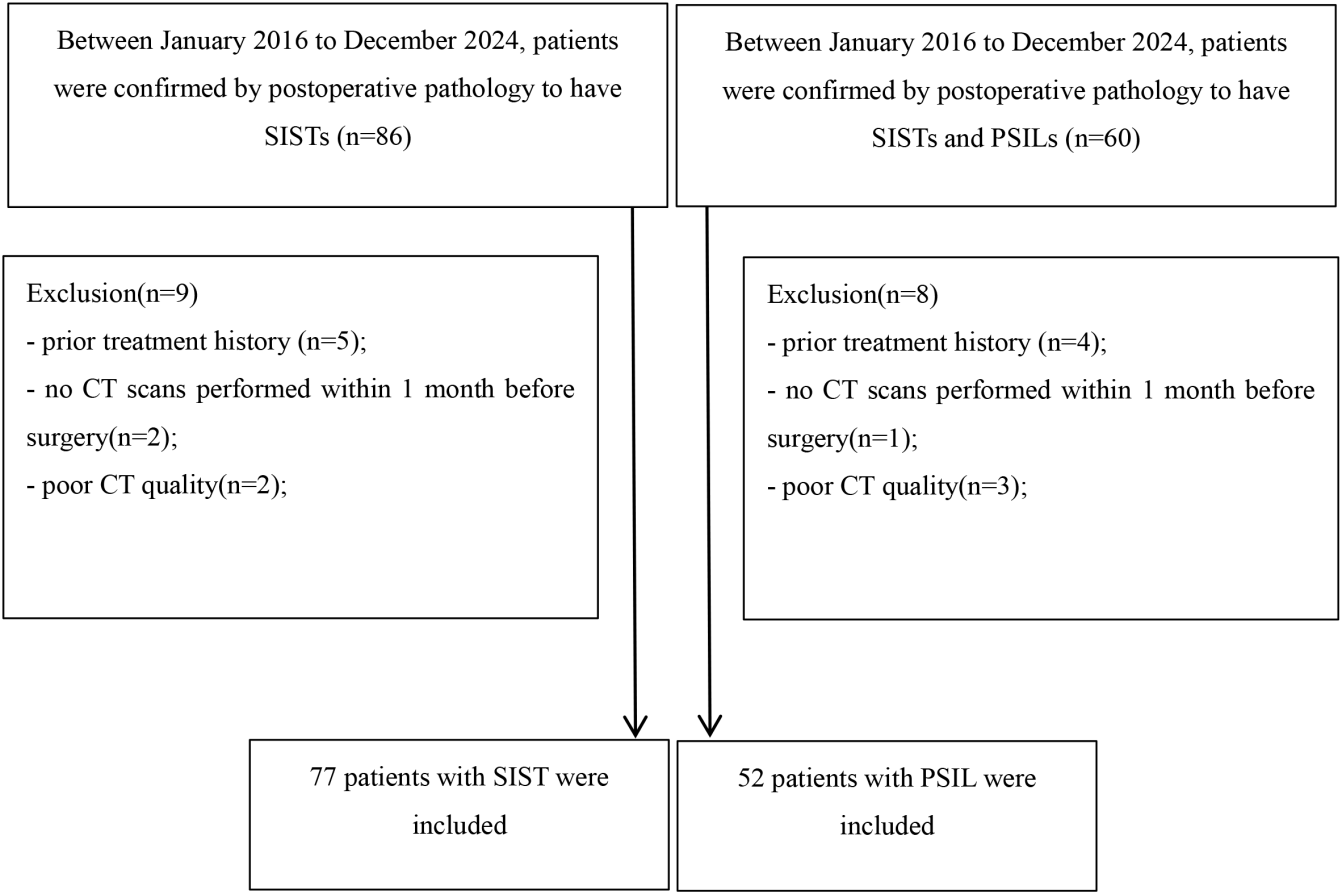
Flow chart detailing the patient selection process and exclusion criteria. In total, 129 patients with small intestinal stromal tumors and primary small intestinal lymphomas were enrolled in the final analysis. SIST, small intestinal stromal tumor; PSIL, primary small intestinal lymphoma;.

This retrospective study was approved by the Institutional Review Board of our hospital, and written informed consent was obtained from all participants. Between January 2016 and December 2024, 146 consecutive patients with pathologically confirmed small intestinal stromal tumors (SISTs) or primary small intestinal lymphomas (PSILs) were enrolled. The inclusion criteria were: (a) curative resection or biopsy with a definitive pathological diagnosis of SIST or PSIL; and (b) availability of contrast-enhanced CT(CECT) performed within 2 months prior to histopathological confirmation. The exclusion criteria were: (a) history of prior treatment (n = 9); (b) absence of preoperative CT within 1 month before surgery (n = 3); and (c) poor-quality CT images due to severe artifacts (n = 5). After applying these criteria, 129 patients (77 SISTs, 52 PSILs) were included in the final analysis. The patient selection process is summarized in **Figure 1**.

### CT Imaging Protocol

All examinations were performed using a dual-source CT scanner (SOMATOM Definition, Siemens Healthcare, Forchheim, Germany). Patients fasted for at least 6 hours prior to scanning and ingested 1500–2000 mL of water 40–60 minutes before the examination to achieve adequate small bowel distension. Scans were acquired in the supine, feet-first position, covering the region from the dome of the diaphragm to the pubic symphysis. The CT acquisition parameters were as follows: tube voltage 100–120 kV, automatic tube current modulation, rotation time 0.5 s, collimation 64 × 1.25 mm, pitch 1.5:1, matrix 512 × 512, and slice thickness/interval 1.25 mm. Images were reconstructed using a 50 cm field of view, standard (STD) kernel, and 100% adaptive statistical iterative reconstruction (ASIR). For contrast-enhanced scans, nonionic iodinated contrast medium (iopromide, 370 mg I/mL; Ultravist 370, Bayer Schering Pharma, Berlin, Germany) was administered intravenously via the cubital vein at 1.5 mL/kg using a high-pressure injector at a rate of 3.5 mL/s, followed by a 20 mL saline flush. Bolus tracking was performed with the region of interest placed in the abdominal aorta, and arterial phase acquisition was initiated 20 s after the attenuation threshold reached 120 HU. Venous phase images were acquired 30s after the completion of the arterial phase, using the same coverage as the unenhanced scan. All images were transferred to a dedicated workstation (Syngovia, Siemens Healthcare) for multiplanar reconstruction with a slice thickness of 1.25 mm in coronal and sagittal planes. Data were subsequently exported to a 3D-Slicer platform for both qualitative and quantitative image analysis.

### Image analysis

All CT images were reviewed on a picture archiving and communication system (PACS) workstation. Two board-certified abdominal radiologists, each with more than five years of experience, independently assessed the images while blinded to clinical information and final pathological results. In cases of disagreement, consensus was reached through consultation with a senior abdominal radiologist with over 30 years of experience. Demographic information such as sex and age were collected. The following imaging characteristics were recorded: lesion contour, margin, location, homogeneity, presence of the embedded vessel sign, luminal expansion, necrosis, lymphadenopathy, and degree of enhancement.

### Feature extraction and selection

**Figure 2:**
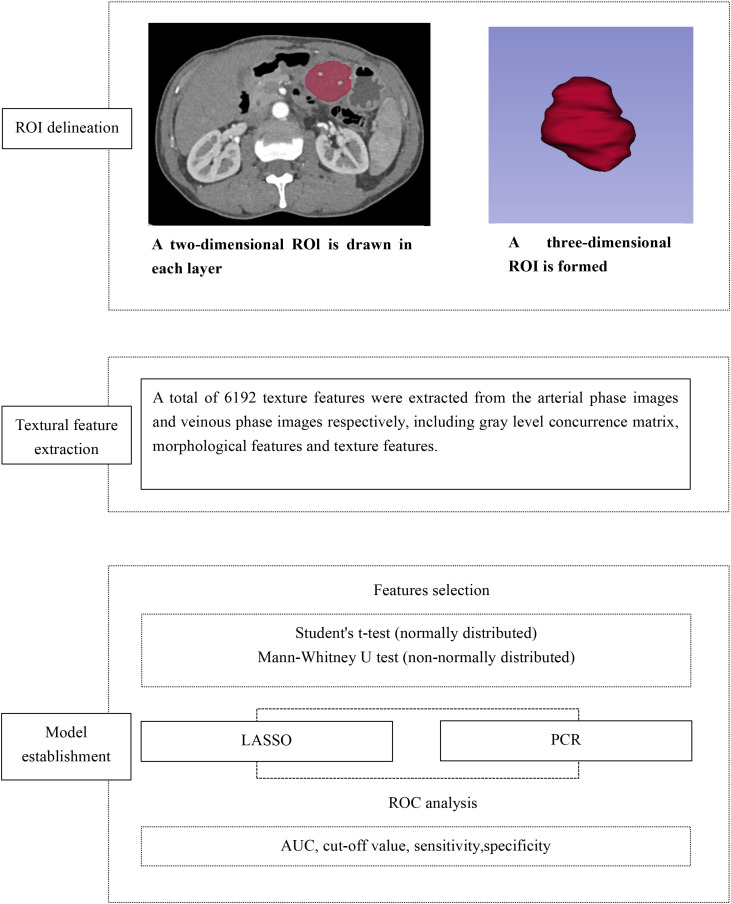
Flow diagram of image processing and texture features calculation.

CT texture features were extracted from arterial- and venous-phase images using 3D Slicer (version 5.6.2; https://www.slicer.org/), an open-source software platform for medical image analysis and visualization (19). The image processing and texture analysis workflow is illustrated in **Figure 2**.

Prior to feature extraction, grayscale normalization was performed to minimize variability caused by differences in image contrast and brightness. The region of interest (ROI) was manually delineated along the tumor boundary on the largest cross-sectional slice by an experienced radiologist blinded to clinical data, except for lesion location. Care was taken to exclude peri-tumoral vessels, adjacent normal bowel wall, intraluminal contents, and surrounding organs. All segmentations were performed on images with a slice thickness of 5 mm to ensure consistency for subsequent analyses.

### Statistical analysis

All statistical analyses were performed using SPSS (version 26.0; IBM Corp., Armonk, NY, USA) and R (version 4.3.1; R Foundation for Statistical Computing, Vienna, Austria). Continuous variables were expressed as mean ± standard deviation or median (interquartile range), depending on distribution, while categorical variables were summarized as counts and percentages. Comparisons between SIST and PSIL groups were performed using the independent-samples t-test or Mann–Whitney U test for continuous variables, and Chi-square or Fisher’s exact test for categorical variables, as appropriate. Univariate analysis was initially conducted to identify statistically significant predictors among CECT morphological features, arterial-phase texture features, and venous-phase texture features. Significant variables were subsequently entered into least absolute shrinkage and selection operator (LASSO) regression for further feature selection.

To develop predictive models, principal component analysis (PCA) was used to reduce dimensionality. Six models were constructed:

CECT morphological features model;

Arterial-phase texture features model;

Venous-phase texture features model;

Combined CECT + arterial-phase texture model;

Combined CECT + venous-phase texture model;

Comprehensive model combining CECT + arterial-phase + venous-phase texture features.

The diagnostic performance of each model in differentiating SISTs from PSILs was evaluated using receiver operating characteristic (ROC) curve analysis. Predictive efficacy was quantified by the area under the curve (AUC), 95% confidence interval (CI), accuracy, sensitivity, and specificity. A two-sided p < 0.05 was considered statistically significant.

## Results

### Patient clinical and morphological characteristics with SIST and PSIL

A total of 129 patients with surgically and pathologically confirmed SISTs or PSILs were included, comprising 77 (59.7%) SISTs and 52 (40.3%) PSILs. The cohort consisted of 83 males and 46 females, with a mean age of 61.51 ± 12.65 years.

Significant differences between the two groups were observed for lesion morphology (p < 0.001), tumor location (p < 0.001),enhanced homogeneity (p < 0.001), vascular embedding (p = 0.011), necrosis (p < 0.001), enlarged lymph nodes (p < 0.001), and enhancement pattern (p < 0.001), margins (p = 0.034) and lumen expansion (p = 0.034). No significant differences were noted in age, sex, or tumor diameter (p > 0.05). The detailed clinical and CT imaging characteristics are summarized in **Table 1**.

**Table 1:** The clinical and CT imaging characteristics of patients.

Parameters		SIST (n=77)	PSIL (n=52)	t/χ²	P
Age (mean ± SD) (y)		63.51 ± 10.65	59.92 ± 16.38	1.391	0.168
Tumor size		64.16 ± 37.26	62.93 ± 28.69	0.200	0.842
Gender	male	46(59.74)	37(71.15)	1.762	0.184
	female	31(40.26)	15(28.85)		
Contour	irregular	23(29.87)	38(73.08)	23.246	<0.001
	regular	54(70.13)	14(26.92)		
Margin	well-defined	67(87.01)	36(69.23)	6.738	0.034
	ill-defined	10(12.99)	16(30.77)		
Location	duodenum	22(28.57)	4(7.69)	37.102	<0.001
	jejunum	30(38.96)	13(25)		
	ileum	25(32.47)	20(38.46)		
	ileocecum	0(0)	15(28.85)		
Homogeneity	homogeneous	11(14.29)	37(71.15)	42.963	<0.001
	heterogeneous	66(85.71)	15(28.85)		
Embedded vessel sign	present	13(16.88)	19(36.54)	6.429	0.011
	absent	64(83.12)	33(63.46)		
Lumen expansion	present	12(15.58)	17(32.69)	4.503	0.034
	absent	65(84.42)	35(67.31)		
Necrosis	present	63(81.82)	9(17.31)	52.377	<0.001
	absent	14(18.18)	43(82.69)		
Lymphadenopathy	present	2(2.6)	35(67.31)	72.446	<0.001
	absent	70(90.91)	17(32.69)		
Enhanced degree	mild	20(25.97)	9(17.31)	62.742	<0.001
	moderate	6(7.79)	38(73.08)		
	obvious	51(66.23)	5(9.62)		

### Arterial-phase CT texture features

Analysis of arterial-phase CT texture features revealed significant differences between SISTs and PSILs in multiple parameters (P < 0.05), including contrast, correlation, difference average, difference variance, energy, entropy, maximum correlation coefficient, joint maximum, sum average, sum entropy, sum of squares, autocorrelation coefficient, cluster prominence, cluster shade, cluster tendency, difference entropy, inverse difference moment (IDM), inverse difference (ID), inverse difference moment normalized (IDMN), inverse difference normalized (IDN), information correlation measures 1 and 2 (ICM1, ICM2), inverse variance, and joint average. These findings indicate distinct arterial-phase texture patterns between the two tumor types. Quantitative comparisons are provided in **Table 2**.

**Table 2:** Comparison of arterial-phase CT texture features.

Parameters	SIST (n=77)	PSIL (n=52)	t	P
Contrast	0.65 ± 0.21	0.36 ± 0.08	11.127	<0.001
Correlation	0.49 ± 0.18	0.27 ± 0.11	8.848	<0.001
Difference average	0.52 ± 0.11	0.35 ± 0.06	10.991	<0.001
Difference variance	0.36 ± 0.09	0.23 ± 0.03	11.557	<0.001
Energy	0.18 ± 0.07	0.35 ± 0.09	-11.478	<0.001
Entropy	3.09 ± 0.51	1.98 ± 0.25	16.424	<0.001
Maximum correlation coefficient	0.53 ± 0.16	0.3 ± 0.11	9.845	<0.001
Joint maximum	0.32 ± 0.1	0.51 ± 0.12	-9.443	<0.001
Sum average	8.42 ± 3.25	7.19 ± 10.74	0.946	0.346
Sum entropy	2.42 ± 0.4	1.59 ± 0.2	15.575	<0.001
SumSquares	0.74 ± 0.4	0.25 ± 0.05	10.673	<0.001
Autocorrelation coefficient	20.77 ± 19.14	41.26 ± 208.56	-0.706	0.483
Cluster prominence	35.09 ± 74.63	1.39 ± 0.84	3.962	<0.001
Cluster shade	0.55 ± 6.55	0 ± 0.18	0.736	0.464
Cluster tendency	2.32 ± 1.5	0.64 ± 0.18	9.703	<0.001
Difference entropy	1.2 ± 0.15	0.94 ± 0.08	12.519	<0.001
IDM	0.76 ± 0.04	0.83 ± 0.03	-10.446	<0.001
ID	0.75 ± 0.05	0.83 ± 0.03	-10.700	<0.001
IDMN	0.99 ± 0.01	0.98 ± 0.01	4.303	<0.001
IDN	0.94 ± 0.02	0.93 ± 0.02	1.511	0.133
ICM1	-0.13 ± 0.08	-0.12 ± 0.08	-0.574	0.567
ICM2	0.49 ± 0.19	0.47 ± 0.19	0.636	0.526
Inverse variance	0.42 ± 0.05	0.34 ± 0.06	8.583	<0.001
Joint Average	4.21 ± 1.63	3.6 ± 5.37	0.946	0.346

IDM, inverse difference moment; ID, inverse difference; IDMN, inverse difference moment normalized; IDN, inverse difference normalized; ICM1, information correlation measure 1; ICM2, information correlation measure 2.

### Venous-phase CT texture features

Venous-phase CT texture analysis similarly demonstrated statistically significant differences between SISTs and PSILs across multiple parameters, including contrast, correlation, and difference average and others. These results highlight distinct venous-phase texture patterns between the two tumor types. Detailed quantitative comparisons are presented in **Table 3**.

**Table 3:** Comparison of venous-phase CT texture features.

Parameters	SIST (n=77)	PSIL (n=52)	t	P
Contrast	0.55 ± 0.18	0.35 ± 0.09	8.011	<0.001
Correlation	0.43 ± 0.18	0.29 ± 0.12	5.265	<0.001
Difference average	0.47 ± 0.11	0.34 ± 0.08	7.804	<0.001
Difference variance	0.31 ± 0.07	0.23 ± 0.04	8.030	<0.001
Energy	0.21 ± 0.07	0.36 ± 0.12	-8.376	<0.001
Entropy	2.78 ± 0.49	1.98 ± 0.34	10.958	<0.001
Maximum correlation coefficient	0.48 ± 0.18	0.32 ± 0.12	6.046	<0.001
Joint maximum	0.41 ± 0.14	0.44 ± 0.15	0.190	0.857
Sum average	7.88 ± 3.65	7.32 ± 11	0.416	0.678
Sum entropy	2.19 ± 0.38	1.6 ± 0.26	10.447	<0.001
SumSquares	0.54 ± 0.27	0.27 ± 0.16	6.946	<0.001
Autocorrelation coefficient	19.09 ± 25.71	43.15 ± 243.46	-0.710	0.481
Cluster prominence	12.63 ± 19.55	471.81 ± 3391.16	-0.976	0.333
Cluster shade	0.15 ± 2	-6.26 ± 44.95	1.027	0.309
Cluster tendency	1.6 ± 1	0.74 ± 0.62	5.999	<0.001
Difference entropy	1.12 ± 0.14	0.93 ± 0.13	7.682	<0.001
IDM	0.78 ± 0.04	0.83 ± 0.04	-7.340	<0.001
ID	0.77 ± 0.05	0.83 ± 0.04	-7.374	<0.001
IDMN	0.98 ± 0.01	0.98 ± 0.01	2.016	0.046
IDN	0.93 ± 0.02	0.94 ± 0.02	-0.580	0.563
ICM1	-0.15 ± 0.08	-0.09 ± 0.05	-5.011	<0.001
ICM2	0.52 ± 0.18	0.34 ± 0.12	6.770	<0.001
Inverse variance	0.41 ± 0.06	0.33 ± 0.07	6.824	<0.001
Joint Average	3.94 ± 1.83	3.66 ± 5.5	0.416	0.678

### Development and diagnostic performance of predictive models

Due to multicollinearity among the statistically significant features and the large number of potential predictors, LASSO regression was initially applied for feature selection. The optimal λ values were as follows: CECT imaging features alone, 0.03942; arterial-phase texture features, 0.00146; venous-phase texture features, 0.02025; combined CECT with arterial-phase features, 0.00133; combined CECT with venous-phase features, 0.00051; comprehensive combination of all features, 0.00212.

**Figure 3:**
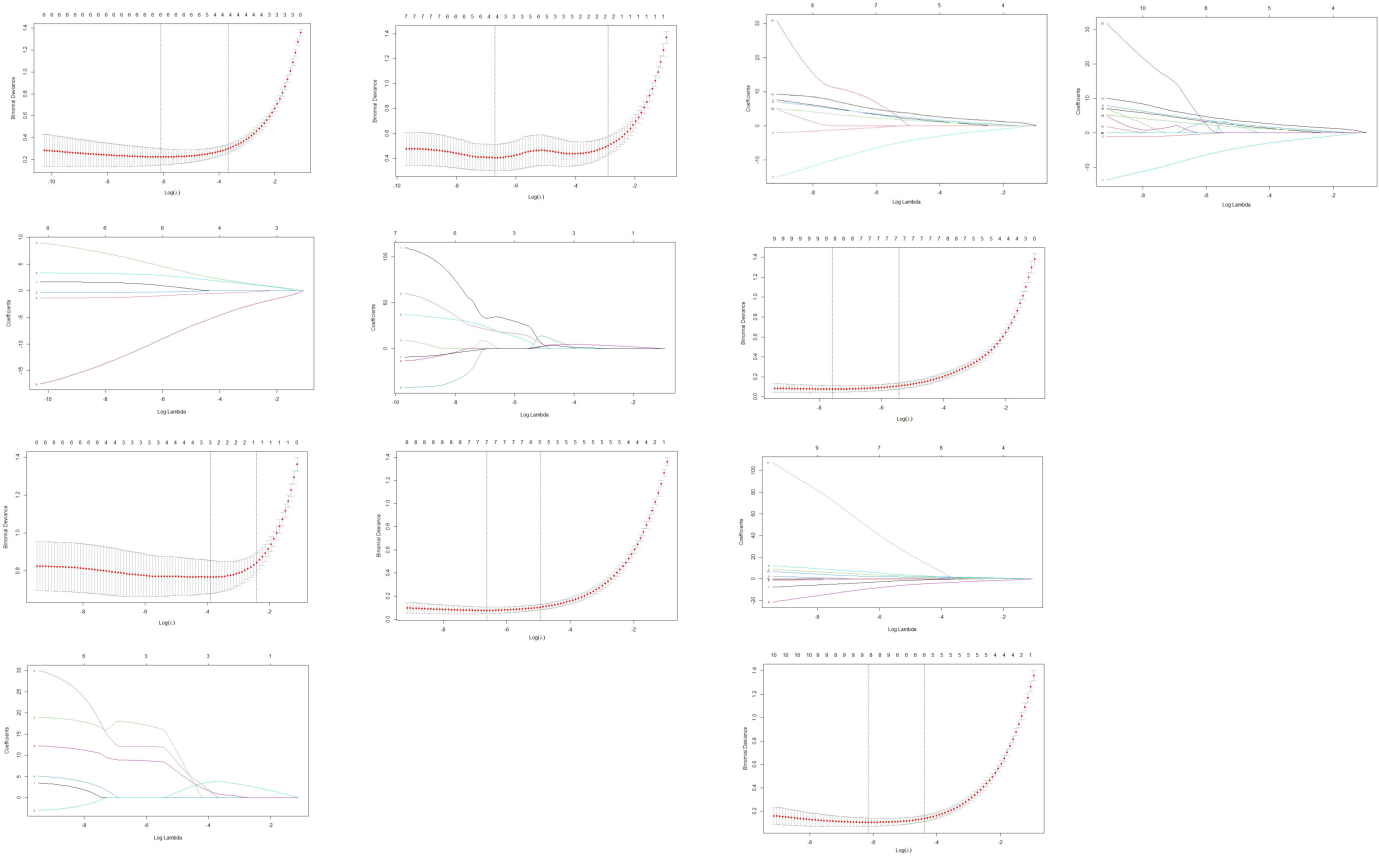
Features selection via LASSO regression. 3-1. Radiomics feature dimensionality reduction via 10-fold cross-validated LASSO regression. 3-2. Radiomics feature coefficient path plot derived from LASSO regression model. 3-3. Venous-phase radiomics feature dimensionality reduction via 10-fold cross-validated LASSO regression. 3-4. Coefficient path plot of venous-phase radiomics features derived from LASSO regression 3-5. Arterial-phase radiomics features dimensionality reduction via 10-fold cross-validated LASSO regression. 3-6. Coefficient path plot of arterial-phase radiomics features derived from LASSO regression model. 3-7. Integrated radiomic and arterial phase features dimensionality-reduction via 10-fold cross-validated LASSO regression. 3-8. Coefficient-path plot of the integrated radiomic and arterial phase features derived from LASSO regression model. 3-9. Integrated radiomic and venousl phase features dimensionality-reduction via 10-fold cross-validated LASSO regression. 3-10. Coefficient-path plot of the integrated radiomic and arterial phase features derived from LASSO regression model. 3-11. Combined-model dimensionality-reduction via 10-fold cross-validated LASSO regression. 3-12. Coefficient-path plot of the combined-model derived from LASSO regression.

As features selected via LASSO regression still exhibited pairwise correlations, principal component regression (PCR) was employed to construct predictive models. Reconstructed regression equations for each model were derived following PCR transformation. The selected features and corresponding regression coefficients are detailed in **Table 4** and **Figure 3**.

**Table 4:** Reconstructed regression equations following Lssso selection and PCR analysis.

Models	optimal λ value	Selection criterion	Regression equation reconstructed after principal-component regression
CECT features	0.03942	homogeneity, enhanced degree, necrosis, lymphadenopathy	Z = 4.616 − 0.599 * (homogeneity) + 9.278 * (enhanced degree) + 1.605 * (necrosis) − 9.867 * (lymphadenopathy)
Arterial-phase CT texture	0.00146	contrast, correlation, mean difference, energy	Z=4.877 + 7.129 * (contrast) + 4.858 * (correlation) + 3.072 * (mean difference) + 4.040 * (energy)
Venous-phase CT texture features	0.02025	difference average, entropy, difference variance	Z = 1.074 + 2.457 * (difference average) + 0.040 * (entropy) + 1.692 * (difference variance)
Combined CECT features with arterial-phase texture features	0.00133	contour, margin, enhanced degree, necrosis, lymphadenopathy, difference variance, entropy	Z =100.889 + 20.369 * (contour) + 14.708 * (margin) + 49.947 * (enhanced degree) + 48.644 * (necrosis) − 45.011 * (lymphadenopathy) + 56.173 * (difference variance) + 60.883 * (entropy)
Combined CECT with venous-phase texture features	0.00051	homogeneity, enhanced degree, embedded vessel sign, necrosis, lymphadenopathy, difference variance, entropy	Z =8.201 − 3.697 * (homogeneity) + 4.323 * (enhanced degree) − 0.474 * (embedded vessel sign) + 3.860 * (necrosis) − 3.859 * (lymphadenopathy) + 5.305 * (difference variance) + 5.721 * (entropy)
Integrated prediction	0.00212	contour, margin, enhanced degree, necrosis, lymphadenopathy, difference variance, entropy, contrast, difference variance	Z=136.455 + 26.880 * (contour) + 21.615 * (margin) +61.441 * (enhanced fegree) + 68.357 * (necrosis) − 58.015 * (lymphadenopathy) + 54.395 * (difference variance) + 62.617 * (entropy) + 26.350 * (contrast) + 28.488 * (difference variance)

CECT Features means CECT morphological features, arterial-phase CT texture means radiomic features extracted from arterial-phase CT images, venous-phase CT texture features means radiomic features extracted from venous-phase CT images, combined CECT with arterial-phase texture features means integration of CECT features with arterial-phase texture features, combined CECT with venous-phase texture features means integration of CECT features with venous-phase texture features, Integrated prediction model means comprehensive combination of CECT features and arterial- and venous-phase texture features.

### ROC analysis of predictive models

**Figure 4:**
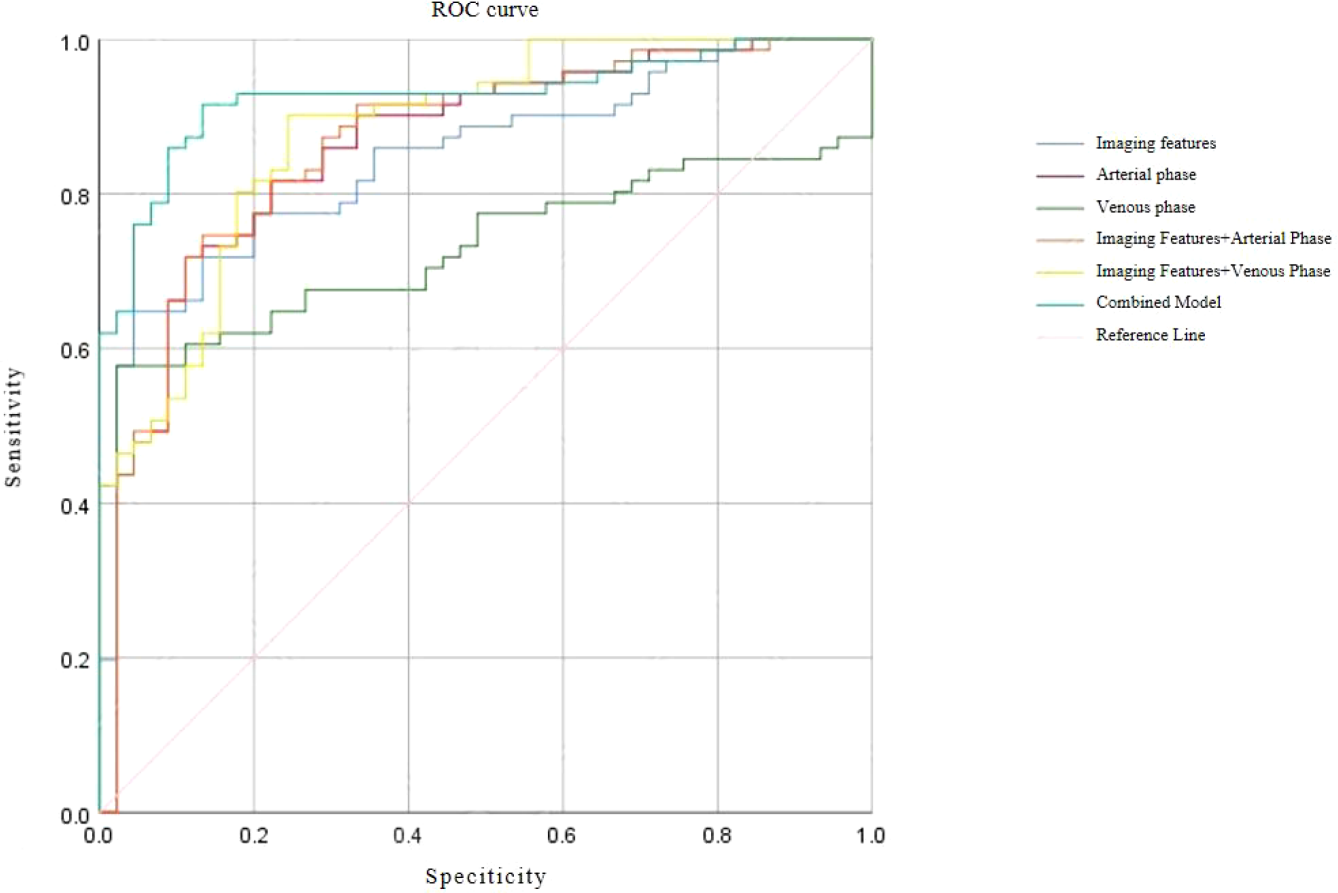
ROC Curve.

Receiver operating characteristic (ROC) curve analysis demonstrated excellent discriminative performance for all predictive models. The area under the curve (AUC) values for each model exceeded 0.9, with sensitivity and specificity also above 0.9, confirming strong diagnostic capability for differentiating SISTs from PSILs. Detailed ROC results and curves are presented in **Table 5** and **Figure 4**.

**Table 5:** Diagnostic performance of predictive models.

Models	AUC(95% CI)	Sensitivity(%)	Specificity(%)
CECT features	0.847 (0.777, 0.917)	0.698	0.906
Arterial-phase CT texture	0.860 (0.790, 0.931)	0.817	0.778
Venous-phase CT texture features	0.731 (0.639, 0.823)	0.707	0.848
Combined CECT features with arterial-phase texture features	0.865 (0.795, 0.935)	0.718	0.889
Combined CECT with venous-phase texture features	0.881 (0.820, 0.943)	0.901	0.756
Integrated prediction	0.927 (0.879, 0.975)	0.915	0.867

## Discussion

In this study, we evaluated the diagnostic performance of CECT combined with texture analysis in differentiating SISTs from PSILs. Multiple CT texture parameters demonstrated significant differences between the two tumor types. Previous studies have highlighted the utility of CT texture analysis in assessing clinical stage, prognosis, and treatment response across various gastrointestinal malignancies, including esophageal cancer (20), gastrointestinal stromal tumors (21, 22), and colorectal cancer (23, 24). Recent study demonstrated that CTTA outperforms conventional clinical and radiologic approaches in distinguishing SISTs from PSILs, and that integrating radiomic features with clinical or imaging data may further optimize predictive accuracy (25). Additionally, quantitative parameters derived from dual-energy spectral CT - such as iodine concentration, virtual monoenergetic imaging (VMI), and effective atomic number (Zeff) - have demonstrated high accuracy in differentiating primary small intestinal tumors (26, 27).

Accurate preoperative differentiation of SISTs from PSILs is clinically important due to distinct treatment strategies. SISTs typically present as exophytic, well-demarcated masses with heterogeneous enhancement resulting from necrosis, hemorrhage, or cystic degeneration. Complications including bleeding, perforation, obstruction, or metastasis can adversely affect the prognosis of patients with SISTs. In contrast, PSILs often manifest as long-segment circumferential wall thickening with homogeneous mild-to-moderate enhancement, preservation of fat planes, and aneurysmal luminal dilatation, frequently accompanied by mesenteric or retroperitoneal lymphadenopathy. Vascular invasion or occlusion is rare, despite frequent vessel encasement. However, overlapping imaging features - including atypical presentations lacking geographic appearance, target signs, aneurysmal dilation, sandwich sign, or floating vessel sign - render differentiation challenging. Conventional CECT diagnostic accuracy ranges from 70-80% for typical SISTs (28), indicating a need for improved diagnostic tools.

Texture analysis provides quantitative assessment of tissue heterogeneity, reflecting tumor microstructure and underlying biological characteristics that are not perceptible by conventional imaging (29). Recent studies have applied CT texture analysis to tumor identification, staging, and therapy response evaluation (30–33), but its application in differentiating SISTs from PSILs has not been previously reported. To our knowledge, this study is the first to employ CT texture analysis for this purpose. High-dimensional radiomic features inherently pose challenges such as multicollinearity and overfitting. To address this, we implemented a two-step dimensionality reduction strategy using LASSO regression followed by Principal Component Analysis (PCA). LASSO effectively identified non-redundant, discriminative features by imposing an L1 penalty, selecting parameters related to heterogeneity and structural complexity - including entropy, contrast, and variance-based measures. PCA subsequently transformed these features into orthogonal principal components (PCs), minimizing multicollinearity while maximizing explained variance. Retained PCs captured key aspects of tumor heterogeneity (entropy, contrast), uniformity (energy, homogeneity), and structural organization (cluster prominence, correlation).

Analysis of arterial-phase features revealed that SISTs exhibited higher contrast, entropy, and inverse difference moment (homogeneity), reflecting complex tissue architecture and intratumoral heterogeneity such as necrosis, hemorrhage, or solid components. Conversely, PSILs showed higher energy and homogeneity, indicating more uniform tissue patterns. Similar trends were observed in the venous phase, with additional parameters - including informational measures of correlation (IMC1 and IMC2) - highlighting differences in spatial organization and grayscale dependence between the two groups. These results support the value of combining LASSO and PCA to derive biologically meaningful and interpretable features from high-dimensional radiomic data.

Our results indicate that CECT imaging features alone can achieve good specificity but limited sensitivity in differentiating SISTs from PSILs. In contrast, arterial- and venous-phase texture analyses demonstrated high pooled sensitivity and specificity. The combined model incorporating imaging and texture features achieved a pooled sensitivity of 91.5% and specificity of 86.7%, underscoring the added value of texture analysis in enhancing diagnostic performance.

This study has several limitations that should be acknowledged. First, this was a single-center retrospective study with a relatively small sample size, the use of a single CT scanner, and a ununiform imaging acquisition protocol, which may introduce selection bias and limit generalizability. Multi-center studies with larger cohorts are needed to validate our findings. Second, while LASSO and PCA enhanced feature selection stability, overall model performance remains constrained by the cohort size. Future studies should externally validate these models across diverse populations. Third, the study lacks both internal and external validation of the developed model. Furthermore, the reliance on manual segmentation, without an assessment of inter-observer reliability or reproducibility, represents another potential source of bias. Finally, although our approach effectively addressed multicollinearity, the performance of alternative techniques (e.g., elastic net regression) was not explored; a comparative analysis of these methods represents an important direction for future research.

## Conclusion

In summary, our findings suggest that CECT combined with texture analysis offers quantitative and reliable parameters for differentiating SISTs from PSILs. This approach enhances preoperative diagnostic accuracy and provides a novel framework for the clinical evaluation and management of intestinal tumors.

## Data Availability

The original contributions presented in the study are included in the article/**Supplementary Material**. Further inquiries can be directed to the corresponding authors.
